# Cerebroprotein hydrolysate attenuates neurodegenerative changes in Alzheimer’s mice model *via* ferroptosis pathway

**DOI:** 10.3389/fphar.2023.1177503

**Published:** 2023-04-19

**Authors:** Moxi Chen, Wei Song, Zhengju Chen, Xiaodong Shi, Xue Wang, Rongrong Li, Honglin Hao, Wei Chen

**Affiliations:** ^1^ Department of Clinical Nutrition, Peking Union Medical College Hospital, Chinese Academy of Medical Sciences and Peking Union Medical College, Beijing, China; ^2^ Medical Science Research Centre, State Key Laboratory of Complex Severe and Rare Diseases, Peking Union Medical College Hospital, Chinese Academy of Medical Science and Peking Union Medical College, Beijing, China; ^3^ Pooling Medical Research Institutes, Hangzhou, China; ^4^ Department of Neurology, Peking Union Medical College Hospital, Chinese Academy of Medical Sciences and Peking Union Medical College, Beijing, China

**Keywords:** Alzheimer’s disease, ferroptosis, oxidative stress, cerebroprotein hydrolysate, APP/PS1 mice

## Abstract

**Introduction:** Cerebroprotein hydrolysate has been proven to improve cognitive function in patients with Alzheimer’s disease (AD). We explored the safety and effectiveness of the clinical administration of oral cerebroprotein hydrolysate in AD, and possible mechanisms related to the neuronal ferroptosis pathway.

**Methods:** Three-month-old male APP/PS1 double-transgenic mice were randomly divided into AD model (n = 8) and intervention (n = 8) groups. Eight non-transgenic wild-type (WT) C57 mice were used as age-matched controls. The experiments were started at the age of 6 months. The intervention group was then administered cerebroprotein hydrolysate nutrient solution (11.9 mg/kg/day) *via* chronic gavage, the other groups received an identical volume of distilled water. Behavioural experiments were performed after 90 days of continuous administration. Serum and hippocampal tissues were then collected for histomorphological observation, tau and p-tau expression, and ferroptosis markers analysis.

**Results:** Cerebroprotein hydrolysate simplified movement trajectories and shortened escape latencies of APP/PS1 mice in the Morris water maze test. Neuronal morphologies were restored in hippocampal tissues on haematoxylin-eosin staining. In the AD-model group, Aβ protein and p-tau/tau expression levels were elevated, plasma Fe^2+^ and malondialdehyde levels were elevated, GXP4 protein expression and plasma glutathione levels declined than controls. All indices improved after cerebroprotein hydrolysate intervention.

**Conclusion:** Cerebroprotein hydrolysate improves learning and memory function, alleviates neuronal damage, and reduces the deposition of pathological AD markers in AD mice, which may be related to the inhibition of neuronal ferroptosis.

## 1 Introduction

Alzheimer’s disease (AD) is a neurodegenerative condition characterised by progressive cognitive impairment and behavioural disorders. The incidence of AD has been increasing annually in rapidly ageing societies (Cui et al., 2020). Gradual exacerbation of AD results in the loss of social function and self-care ability, which in turn impose a great burden on the families of patients and society in general. At present, cholinesterase inhibitors, which can delay the loss of cognitive function, constitute the principal pharmacotherapy for the management of AD. However, their long-term administration results in various adverse effects such as anorexia, weight loss, and bradycardia (Rabins et al., 2017). Nutrients such as vitamins, polyphenols, and polyunsaturated fatty acids that can improve the oxidative stress states of nerve cells have been found to possess strong potential to alleviate the neurodegenerative process of AD (Ravi et al., 2019). However, the specific mechanisms and pharmacological targets of these agents remain to be clarified.

Recent studies have found a close association between oxidative stress in nerve cells and ferroptosis (Zhao, 2019), a pathway of programmed cell death that is distinct from apoptosis, autophagy, and other mechanisms. Ferroptosis is an iron-dependent non-apoptotic cell necrosis, which is characterised by intracellular iron overload and lipid peroxidation. It is mainly associated with a reduction in the levels of glutathione (GSH) and glutathione peroxidase (GPXs) expression (Vitalakumar et al., 2021). Nutritional supplements related to GSH synthesis and intracellular oxidative stress states may improve the inflammatory state of nerve cells by regulating the ferroptosis-related pathway (Ashraf and So, 2020).

Cerebroprotein hydrolysate is a widely used neurotrophic supplement and has been shown to effectively improve neuronal oxidative stress in AD models (Patocková et al., 2003). It is an aqueous solution of a mixture of various amino acids and low-molecular-weight peptides obtained by the enzymatic hydrolysis of animal brain tissue. Previous studies have shown that cerebroprotein hydrolysate can reduce the formation and phosphorylation of amyloid in neurons by regulating the activities of glycogen synthase kinase 3β (GSK3β) and cyclin-dependent kinase 5 (Rockenstein et al., 2006). As an important target in regulating oxidative stress and programmed cell death, GSK3β also was found to play a vital role in the process of ferroptosis of nerve cells (Wang et al., 2021). Thus, the neuroprotective function of cerebroprotein hydrolysate may be achieved by inhibiting ferroptosis.

Several clinical studies have proved that parenteral administration of cerebroprotein hydrolysate can effectively enhance cognitive function, memory, and activities of daily living of patients with AD, and consequently, improve their quality of life (Alvarez et al., 2011). However, because of the shortcomings of intravenous administration, such as the need for professional operation and more complications, this study aimed to verify the safety and effectiveness of the clinical administration of oral cerebroprotein hydrolysate. And, to explore whether the effect of cerebroprotein hydrolysate in improving oxidative stress in nerve cells (and ultimately the outcomes of AD) is related to the ferroptosis pathway in nerve cells.

## 2 Materials and methods

### 2.1 Animals and administration

APP/PS1 double-transgenic mice were purchased from Jicui Yaokang Animal Experiment Center (Nanjing, China). With reference to previous designs of APP/PS1 double-transgenic mice in Morris water maze test (Huang, Li et al., 2017; Che, Li et al., 2018; Chen, Wang et al., 2022), sixteen 3-month-old male mice were randomly divided into two groups as follows: 1) AD model group (model group, n = 8), 2) AD model + cerebroprotein hydrolyzate group (intervention group, n = 8), and eight non-transgenic wild-type (WT) C57 mice (control group, n = 8) were used as age-matched controls. The experiments were started at the age of 6 months. The cerebroprotein hydrolysate solution used in this study was obtained from Heimerkang Brain Polypeptide (Zhitong Biopharmaceutical Co., Ltd., Hebei, China).

From the age of 6 months, the intervention group was administered cerebroprotein hydrolysate 11.9 mg/kg/day of body weight (calculated based on the Human Equivalent Dose (Boxenbaum et al., 1995), cause the body surface area of a person weighting 70 kg is 378.9 times that of a mice weighting 20 g, and the cerebroprotein hydrolysate administration for human is 1.28 mg/kg/day) *via* chronic gavage. The model and control groups were administered distilled water at the same volume. Behavioural experiments were performed after 90 days of continuous administration. Before sacrifice, anaesthesia was induced, brain tissues and the serum of the retrobulbar venous plexus were collected from each mouse for subsequent analysis. Animal welfare guidelines of this study abided by China Laboratory Animal Welfare Law and Animal management regulations, Number: GB/T 35,892-20181, and the experimental protocol was approved by the Ethics Committee for the Welfare of Laboratory Animals of Peking Union Medical College Hospital (No. XHDW-2019-008). The tenets of the Declaration of Helsinki and the National Institutes of Health guidelines for the use of experimental animals were strictly followed during the research process.

### 2.2 Morris water maze

The Morris water maze (MWM) was used to observe the effect of cerebroprotein hydrolysate on spatial learning and memory abilities of the mice. The apparatus consisted of a circular water-filled pool 150 cm in diameter, 50 cm in height, and with a water temperature of 22°C–24°C, which was divided into four equal quadrants. A cylindrical platform measuring 10 × 10 cm was constructed in one of the quadrants, the target quadrant. The top of the platform was located 1 cm below the water level. The other quadrants were non-target or peripheral quadrants. For each test, mice were placed in the water in a random quadrant and allowed to swim freely for 60 s. The experiment was divided into three periods. On the first day of the test, all mice underwent training, learning, and memory exercises with the platform visible. Mice with disorders of physical function or movement were excluded. On days 2–6, concealed platform experiments were conducted to test the learning and memory abilities of the mice: white dye was added to the water to obscure the platform and the animal’s ability to find the platform was tested. If the platform was found within 60 s, the animal was allowed to rest on the platform for 10 s. If the platform was not found within 60 s, the animal was guided to the platform, and allowed to stay there for 10 s to enhance memory. The test and training exercises were repeated four times a day. On the 7th day of the experiment, the animal’s memory retention ability was assessed in the spatial exploration experiment. The platform was removed from the tank 24 h after the end of the concealed platform experiment. Thereafter, a quadrant was randomly selected and the animal was placed into the water; the swimming path and the number of times the location of the original target platform was traversed within 60 s were recorded. A computerized video tracking system (Supermaze Morris; Shanghai Xinruan Information Technology Co., Ltd., Shanghai, China) recorded the path latency and calculated the swimming time as previously described (Wang et al., 2018).

### 2.3 Histomorphological changes

Morphological changes in the hippocampus were observed using haematoxylin-eosin (HE) staining. Briefly, when the hemisphere (the left brain) were formalin-fixed, the middle (sagittal plane) of the approximate 4 mm of the brain were collect to be dehydrated, embedded, sectioned in paraffin for routine HE staining and histopathological analysis. Light microscope (Nikon, Japan) was used for observation and photographed at 200 × magnification. The whole hippocampal regions were observed by staining after the section, and the most significant part of the differences was demonstrated in the Figures and kept in the same field of vision among the model, intervention and control group.

### 2.4 Western blot

Western blot (WB) tests were conducted to analyse protein expression in hippocampal tissues. The tissues were soaked in single-detergent lysis buffer containing phenylmethanesulfonyl fluoride and phosphatase inhibitors. After ultrasonic homogenisation, the sample was placed on ice for 30 min to enable complete lysis, followed by centrifugation at 12,000 rpm and 4°C for 5 min, and the resultant supernatant was collected. Protein concentrations were determined using the bicinchoninic acid (BCA) assay.

Equal amounts of protein were loaded into each well and subjected to sodium dodecyl sulphate polyacrylamide gel electrophoresis, and the separated proteins were transferred to a polyvinylidene fluoride (PVDF) membrane (MERCK, Germany). The PVDF membranes were soaked in tris-buffered saline with Tween (blocking solution) containing 5% non-fat dry milk, blocked with a shaker at room temperature for 2 h, and each membrane was treated with the following specific primary antibodies at 4°C: mouse anti-Aβ (1:1000, Affinity, AF6084), anti-tau (1:1000, Affinity, AF6141), anti-p-tau (1:1000, Affinity, AF3148), *β*-actin (43KD) (1:5000, Affinity Biosciences, AF7018), and mouse anti-GPX4 (1:1000, Abcam, ab125066). HRP-labelled goat anti-rabbit secondary antibody (Wuhan Boster Biological Engineering Co., Ltd., BA1054) was incubated at room temperature for 2 h. The immunoreactive bands were visualised using enhanced chemiluminescence reagents and grayscale analysis was performed using BandScan software.

### 2.5 Ferroptosis markers

The Fe^2+^ levels were measured from mouse plasma using the Iron Assay Kit (JaICA/CFE-005) according to the manufacturer’s instructions. GSH levels were measured using the GSH kit (Nanjing Jiancheng Bioengineering Institute, A005-1-2). The malondialdehyde (MDA) Kit (Nanjing Jiancheng Bioengineering Institute, A003-1-2) was used to measure the levels of the lipid peroxidation product MDA, while the reactive oxygen species (ROS) Kit (Nanjing Jiancheng Bioengineering Institute, E004) was used to measure the intracellular lipid ROS level.

### 2.6 Statistical analysis

All data were presented as the mean ± standard error mean (SEM). Differences between two groups were analysed using t-tests, and one-way analysis of variance (ANOVA) was employed for multiple comparisons. All data were analysed using Graphpad Prism 6.01 software. *p*-values below 0.05 were considered statistically significant.

## 3 Results

### 3.1 Cerebroprotein hydrolysate improves AD symptoms in APP/PS1 transgenic mice

**FIGURE 1:**
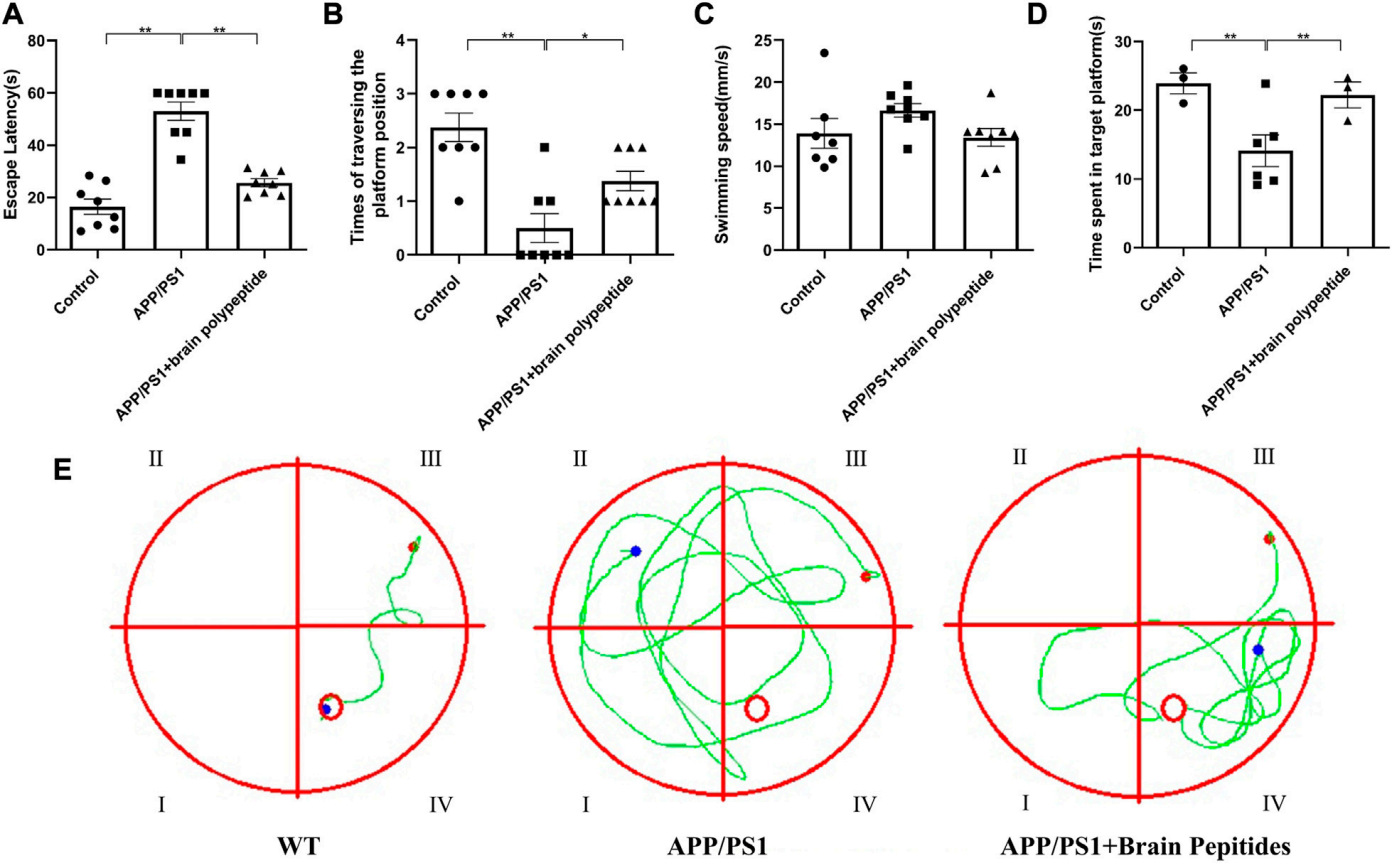
Cerebroprotein hydrolysate improves the learning and memory ability of APP/PS1 mice. (n = 8). Note: **(A)** escape latency of mice in each group; **(B)** number of times the mice traversed the platform in each group; **(C)** swimming speed of mice in each group; **(D)** number of times the mice entered the target quadrant in each group; **(E)** swimming trajectory of the mice in each group. The data are expressed as the mean ± standard error mean ( $\bar{x}\pm \mathrm{sem}$ ), as revealed by the one-way analysis of variance. *p < 0.05, **p < 0.01.

In the MWM test, compared with the control group, the escape latency of APP/PS1 transgenic mice were increased, the times of traversed the platform and the time of staying in the target quadrant were shortened. While the escape latency of intervention group were shorter than that of the model group, and the time of staying in the target quadrant were also increased (Figures 1A–D). The swimming trajectory of mice showed that the track of the control group was clear and the distance was short, while the APP/PS1 transgenic mice had a wide range of movement tracks and could not find the platform accurately and quickly. After the administration of cerebroprotein hydrolysate, the swimming paths of APP/PS1 transgenic mice, became clearer and shorter than those of the model group (Figure 1E).

### 3.2 Cerebroprotein hydrolysate improves pathological changes in APP/PS1 transgenic mice

**FIGURE 2:**
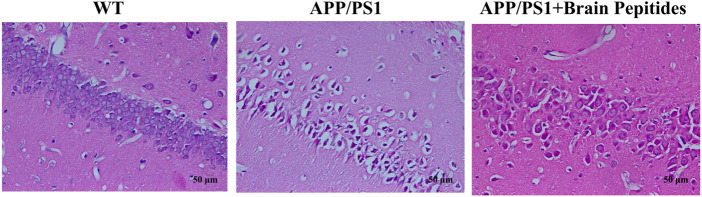
Cerebroprotein hydrolysate improves the morphology and number of hippocampal neurons in APP/PS1 mice (haematoxylin-eosin staining, × 200) (n = 3).

**FIGURE 3:**
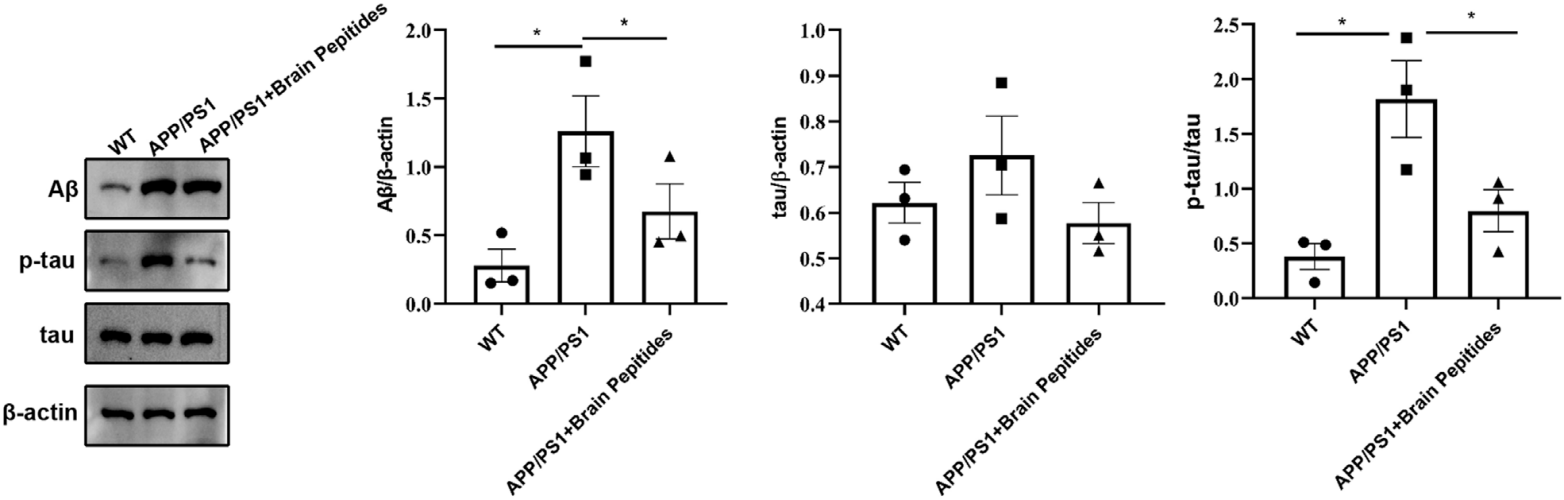
Cerebroprotein hydrolysate improves the expression of Aβ, tau and p-tau in the hippocampus of APP/PS1 mice; (n = 3). Note: The data are expressed as the mean ± standard error mean ( $\bar{x}\pm \mathrm{sem}$ ), as revealed by the one-way analysis of variance. *p < 0.05, **p < 0.01.

At the morphological level, the hippocampal neurons of the model group were deformed, the nuclei appeared pyknotic and the neurons were loosely arranged, along with structural disorganisation. The morphology of the nucleus and cytoplasm was recovered in the neurons of the intervention group, the nuclei stained blue, the neurons were densely packed (Figure 2). The results of WB showed that brain Aβ protein and p-tau/tau ratio were downregulated in APP/PS1 mice compared to the model group after the administration of cerebroprotein hydrolysate (Figure 3).

### 3.3 Ferroptosis-related biological markers in APP/PS1 transgenic mice

**FIGURE 4:**
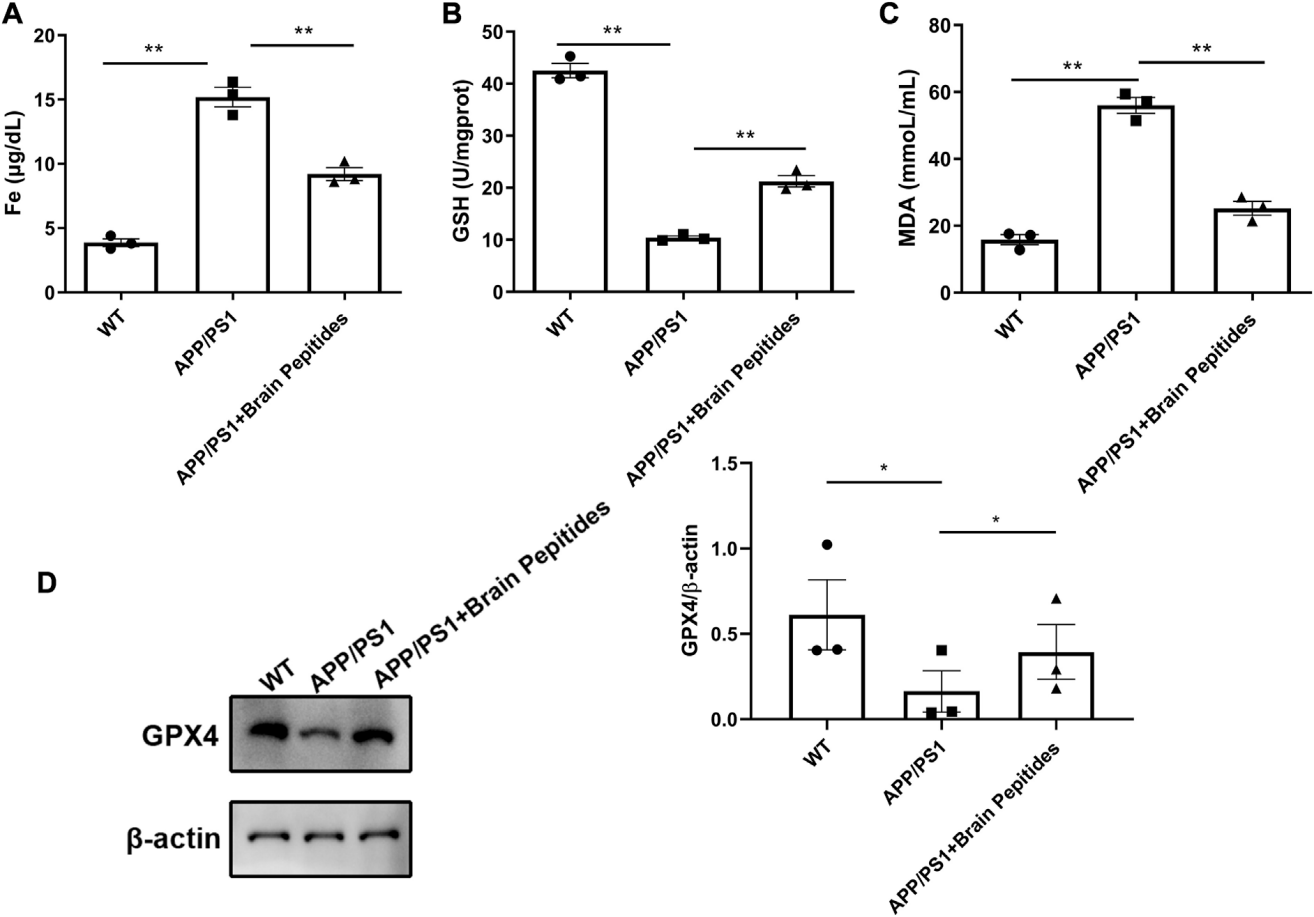
**(A–C)** Cerebroprotein hydrolysate regulates plasma iron, MDA, and GSH levels in APP/PS1 mice; (n = 3) **(D)** Cerebroprotein hydrolysate upregulates the expression of GPX4 protein in the hippocampus of APP/PS1 mice; (n = 3). Note: The data are expressed as the mean ± standard error mean ( $\bar{x}\pm \mathrm{sem}$ ), as revealed by the one-way analysis of variance. *p < 0.05, **p < 0.01; GPX4: glutathione peroxidase, GSH: glutathione, MDA: malondialdehyde.

The plasma iron level of APP/PS1 transgenic mice was higher than that of the control group, but decreased with administration of cerebroprotein hydrolysate (Figure 4A). The levels of GSH and lipid peroxide MDA were also reduced (Figures 4B, C). The results of WB showed that GXP4 protein in the hippocampus of the mice in the APP/PS1 group was downregulated compared to the control group (Figure 4D).

## 4 Discussion

Our study confirmed that oral cerebroprotein hydrolysate can effectively improve the learning and memory function of AD mice, delay neuronal damage in the hippocampus, limit the accumulation of Aβ protein, and downregulate the p-tau/tau ratio. This therapeutic effect is closely related to the regulation of the neuronal ferroptosis pathway, i.e., upregulation of GSH and GPX4 protein expressions in the hippocampus, downregulation of plasma iron levels, and reduction of lipid peroxidation and ROS levels, thereby increasing cell viability.

APP/PS1 double transgenic mouse is one of the classic AD models with typical pathological changes. In previous study, it has been found that there is iron overload in the cortex and hippocampus of APP/PS1 mice (Li et al., 2019). And iron overload in neurons is an important characteristic of ferroptosis, which is a recently discovered iron-dependent, oxidative, non-apoptotic cell death pathway. Ferroptosis is believed to be closely related to the changes in intracellular antioxidant GSH concentration (Zhang et al., 2021), the level of GPXs expression (Yoo et al., 2010), and ultimately manifested as cell death induced by lipid peroxidation accumulation. We found that the Fe^2+^ concentration was elevated and GSH and GPX4 levels were reduced in the hippocampus of AD mice, which is consistent with the increase in lipid peroxide MDA concentration (Forcina and Dixon, 2019) and the damage to neuronal function (Zhu et al., 2022). These changes confirmed the important role of ferroptosis in neuronal cell death in AD mice (Morris et al., 2018).

The treatment methods targeting intracellular iron overload and lipid peroxidation (Chen et al., 2021), such as deferoxamine (Guo et al., 2013), desferrioxamine (Crapper McLachlan et al., 1991) and N-acetylcysteine (Andrade et al., 2020), have shown potential role in slowing down the clinical progression of AD-related dementia in animal models and clinical trials. This therapeutic effect may be achieved by targeting the GSH/GPX4 axis (More et al., 2018) in ferroptosis pathway, and is closely related to the oxidative state in cells. Our study suggested that cerebroprotein hydrolysates that can ameliorate neuronal oxidative stress and improve intracellular GSH levels (Alzoubi et al., 2020) may also delay the progression of AD by targeting ferroptosis pathway.

Previous studies have suggested that cerebroprotein hydrolysate exerts multi-target effects on the pathophysiological processes of AD and can function as an endogenous neurotrophic factor (Gavrilova and Alvarez, 2021). These pathophysiological processes include the deposition of characteristic pathological proteins (Aβ and tau) in the hippocampus, neuroinflammation and oxidative stress. Our study suggested that cerebroprotein hydrolysates also played an important role in improving ferroptosis in nerve cells, and the potential mechanism of inhibiting ferroptosis-related peroxidation. These findings were consistent with previous research that cerebroprotein hydrolysate can effectively improve oxidative stress and reduce lipid peroxidation in models of nerve damage induced by hypoglycaemia (Patocková et al., 2003), 3-nitropropionic acid (Calderón Guzmán et al., 2016), chronic alcoholism (Vaghef et al., 2019), etc.

This study also has some limitations. Morris water maze is the most commonly used behavioral test method to evaluate the learning and memory in APP/PS1 model, but somewhat stressful. More and milder behavioral experimental methods like the Barnes maze should be introduced in the future studies. In the histomorphological analysis, we observed the whole hippocampal regions rather than by different areas (CA1-CA4, DG), and we will show multiple locations of a hippocampus in the further research. This is a preliminary study to determine the feasibility of targeting ferroptosis in AD therapy, so we only observed the changes of ferroptosis related markers before and after intervention. In future research, we will continue to explore the targeted sites of AD treatment through ferroptosis pathway.

## 5 Conclusion

Cerebroprotein hydrolysate improves learning and memory function, alleviates neuronal damage, and reduces the deposition of AD pathological markers in AD mice. This therapeutic effect may be related to the inhibition of oxidative stress and neuronal ferroptosis. Ferroptosis may be a potential target for the treatment of neuronal degenerative diseases in the future.

## Data Availability

The original contributions presented in the study are included in the article/Supplementary Material, further inquiries can be directed to the corresponding authors.
