# Supplementary figures and images for: Cerebroprotein hydrolysate attenuates neurodegenerative changes in Alzheimer’s mice model via ferroptosis pathway
Source: Front Pharmacol. 2023 Apr 19;14:1177503. doi: 10.3389/fphar.2023.1177503 (PMC10154667; doi:10.3389/fphar.2023.1177503)

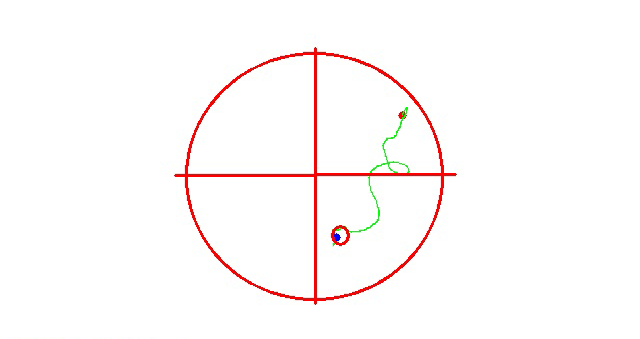

Supplement: Supplementary file 1 [file DataSheet1.ZIP › Original data/Figure 1 Morris/trajectory-control.tif]

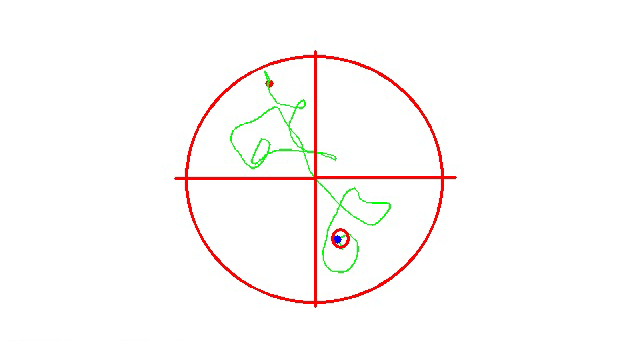

Supplement: Supplementary file 1 [file DataSheet1.ZIP › Original data/Figure 1 Morris/trajectory-intervention.tif]

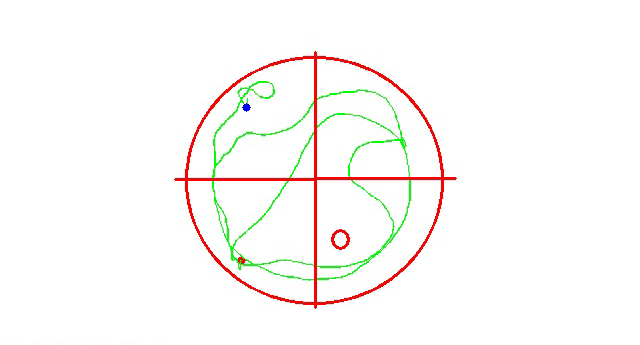

Supplement: Supplementary file 1 [file DataSheet1.ZIP › Original data/Figure 1 Morris/trajectory-model.tif]

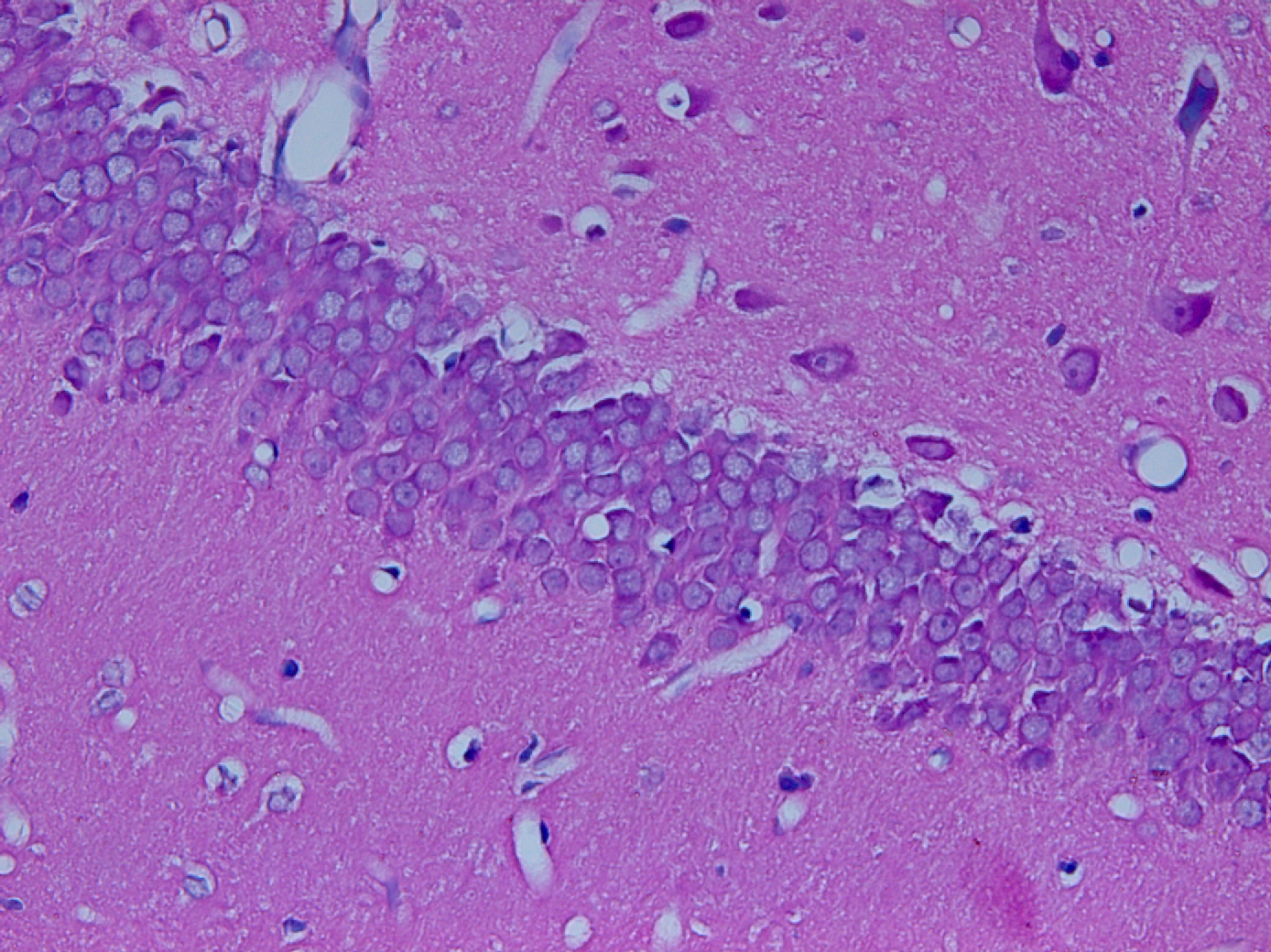

Supplement: Supplementary file 1 [file DataSheet1.ZIP › Original data/Figure 2 HE/HE-control.jpg]

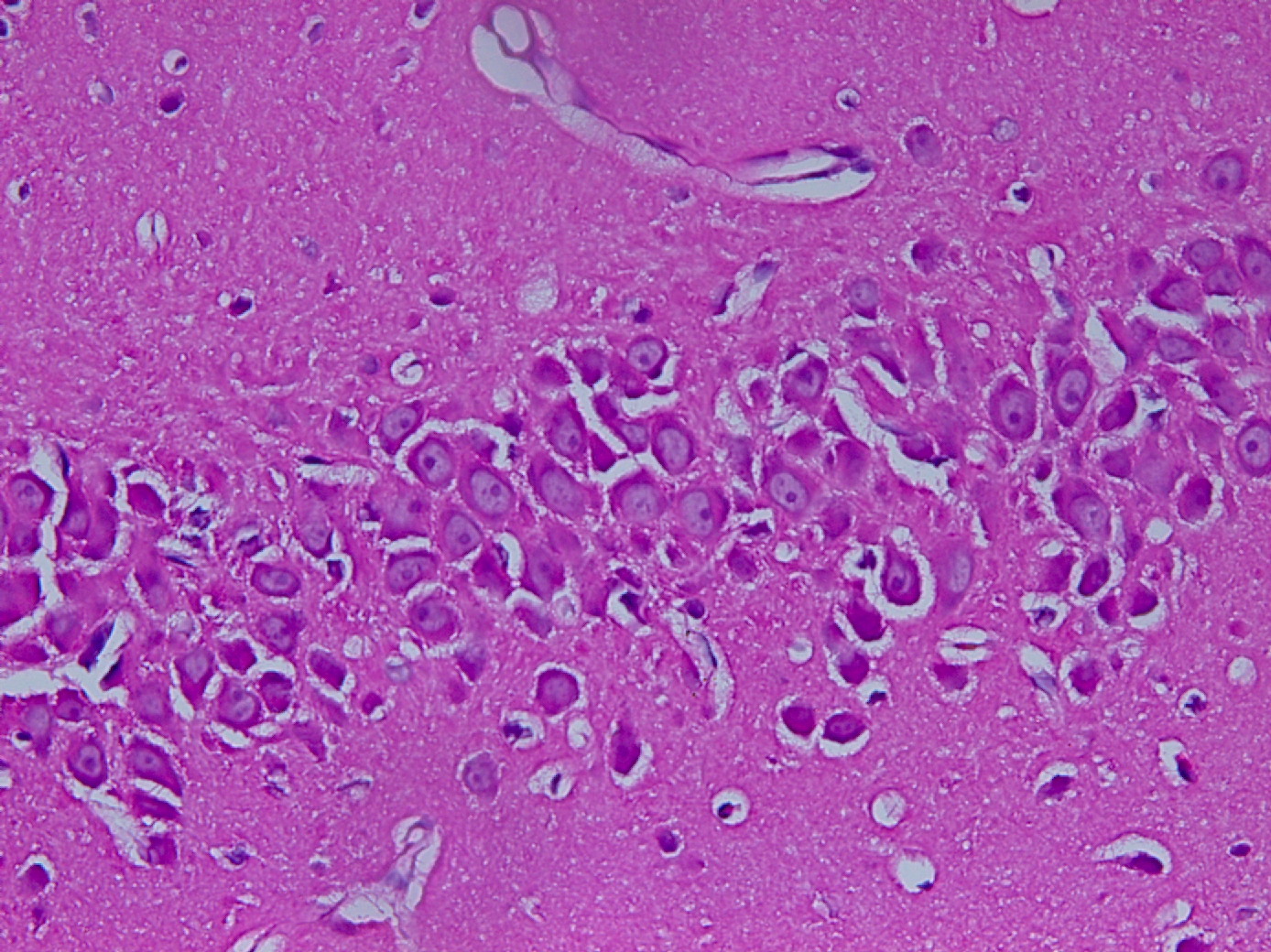

Supplement: Supplementary file 1 [file DataSheet1.ZIP › Original data/Figure 2 HE/HE-intervention.jpg]

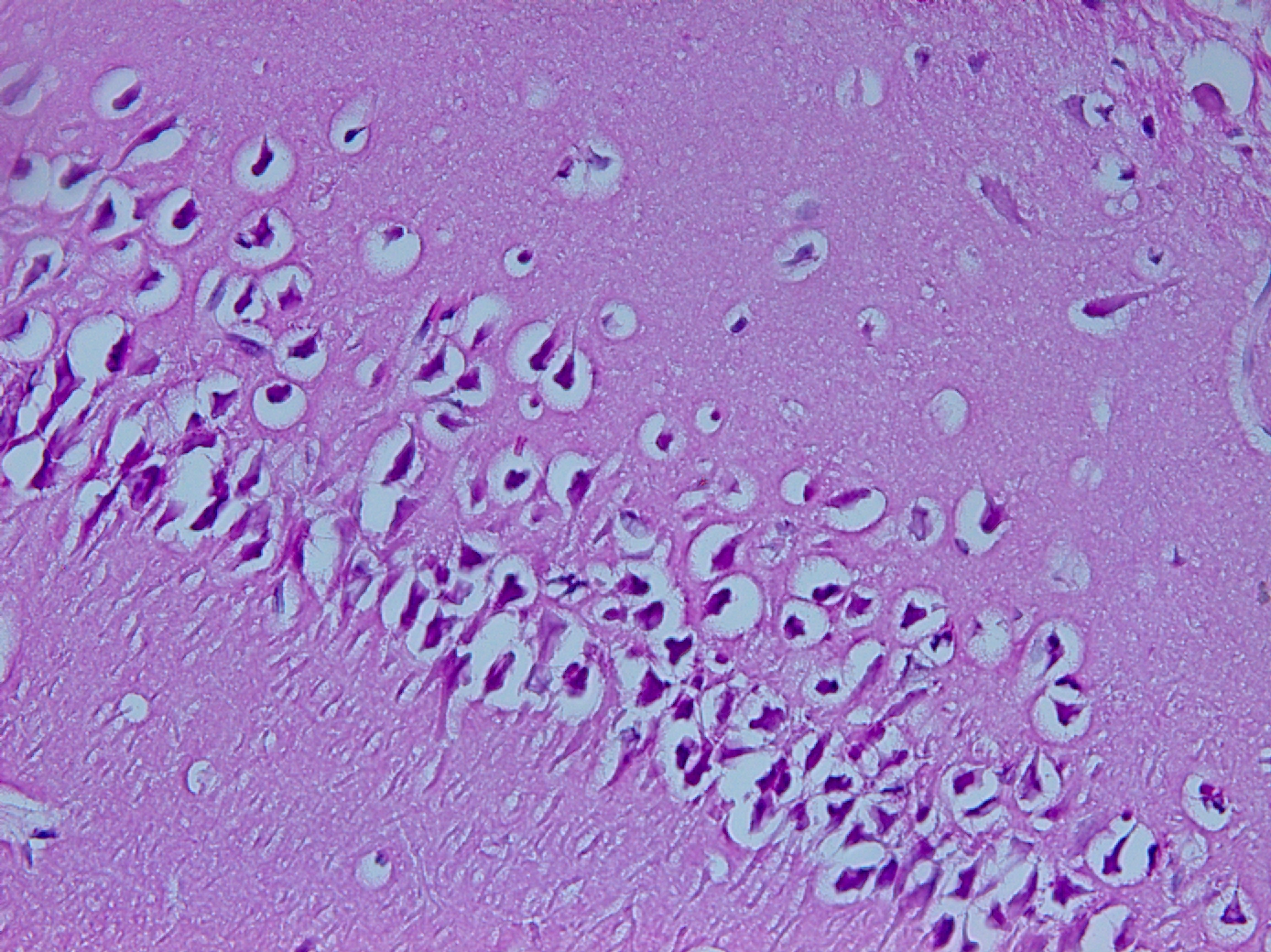

Supplement: Supplementary file 1 [file DataSheet1.ZIP › Original data/Figure 2 HE/HE-model.jpg]

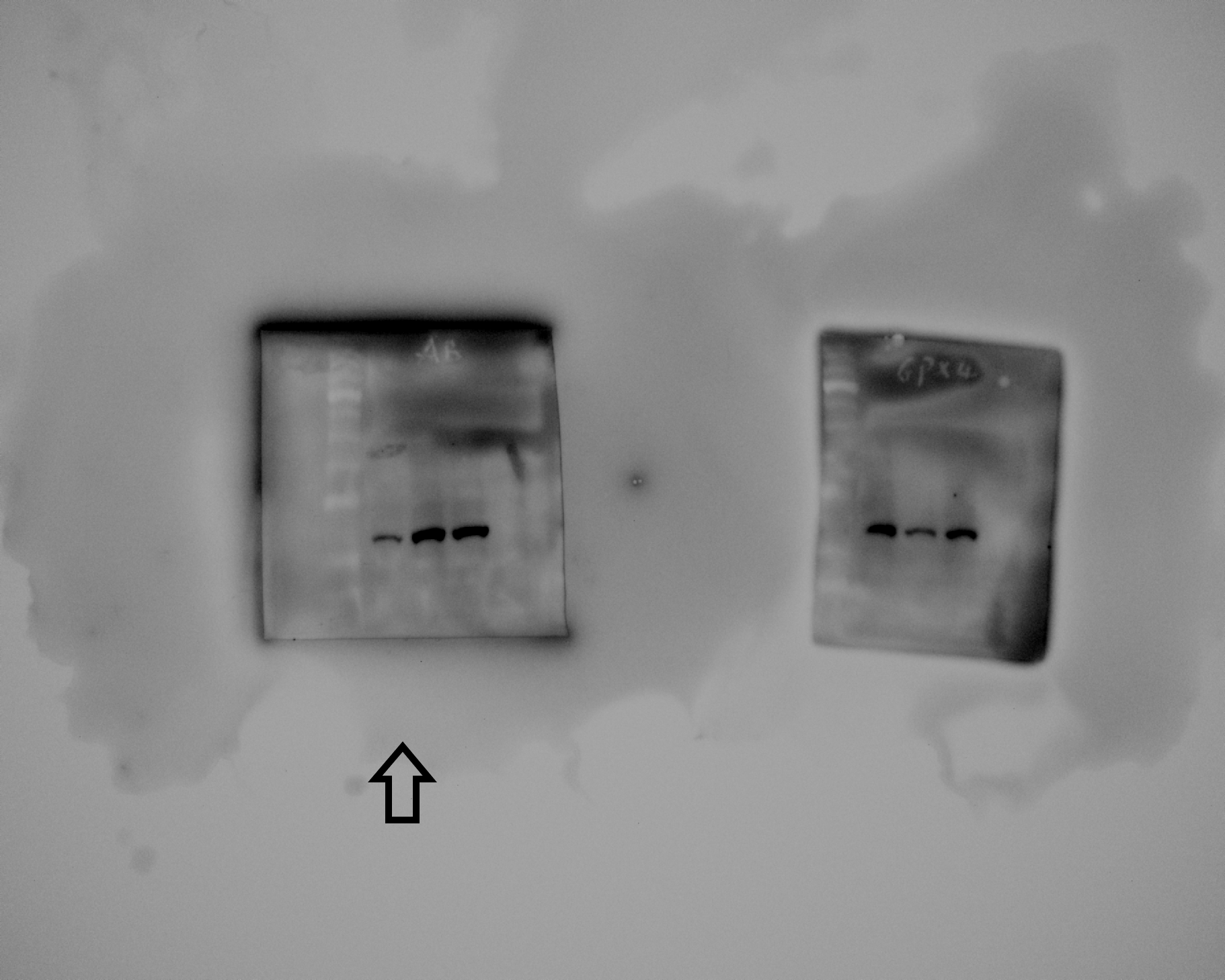

Supplement: Supplementary file 1 [file DataSheet1.ZIP › Original data/Figure 3 Aa┬, tau, p-tau/Aa┬.tif]

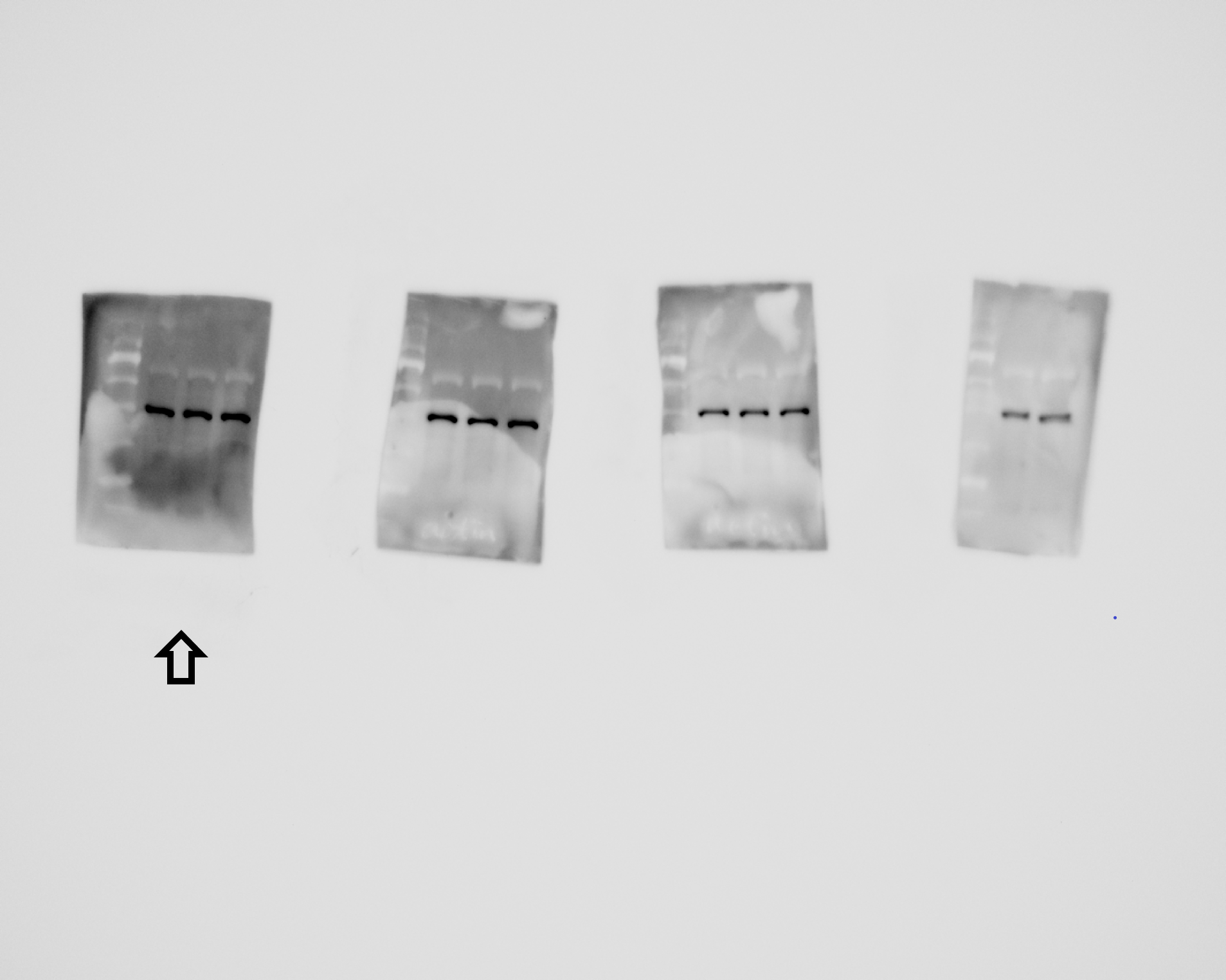

Supplement: Supplementary file 1 [file DataSheet1.ZIP › Original data/Figure 3 Aa┬, tau, p-tau/actin.tif]

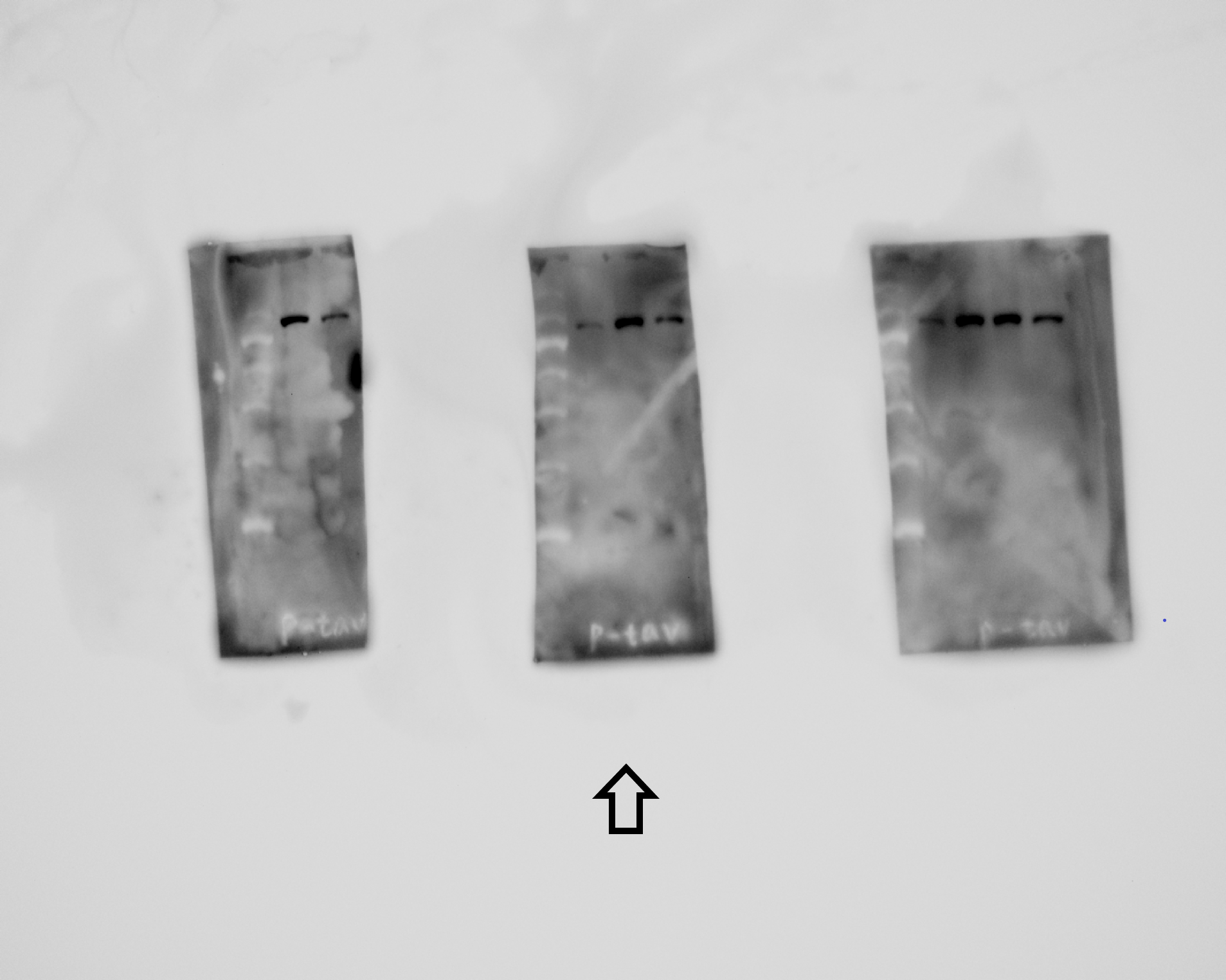

Supplement: Supplementary file 1 [file DataSheet1.ZIP › Original data/Figure 3 Aa┬, tau, p-tau/p-tau.tif]

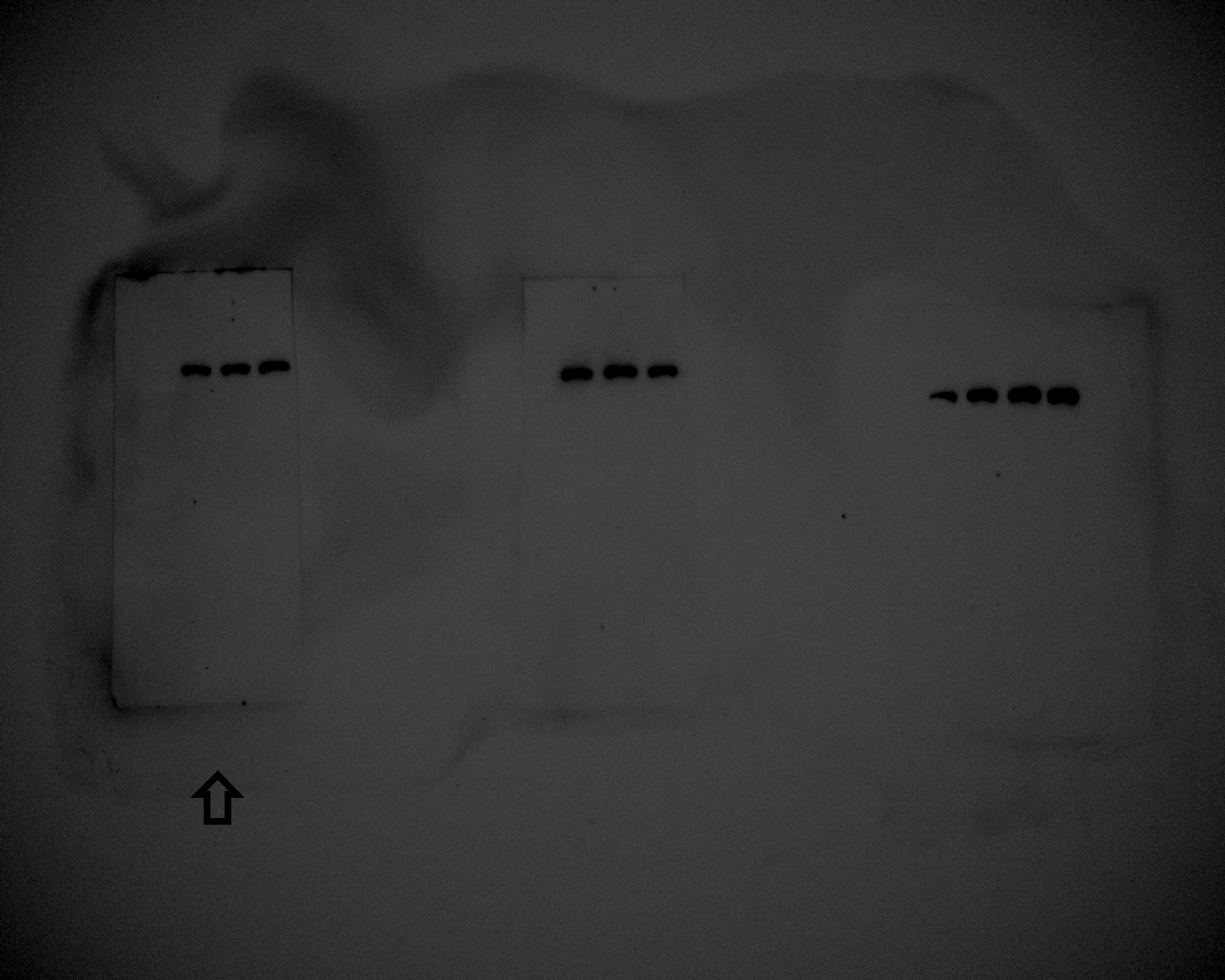

Supplement: Supplementary file 1 [file DataSheet1.ZIP › Original data/Figure 3 Aa┬, tau, p-tau/tau.tif]

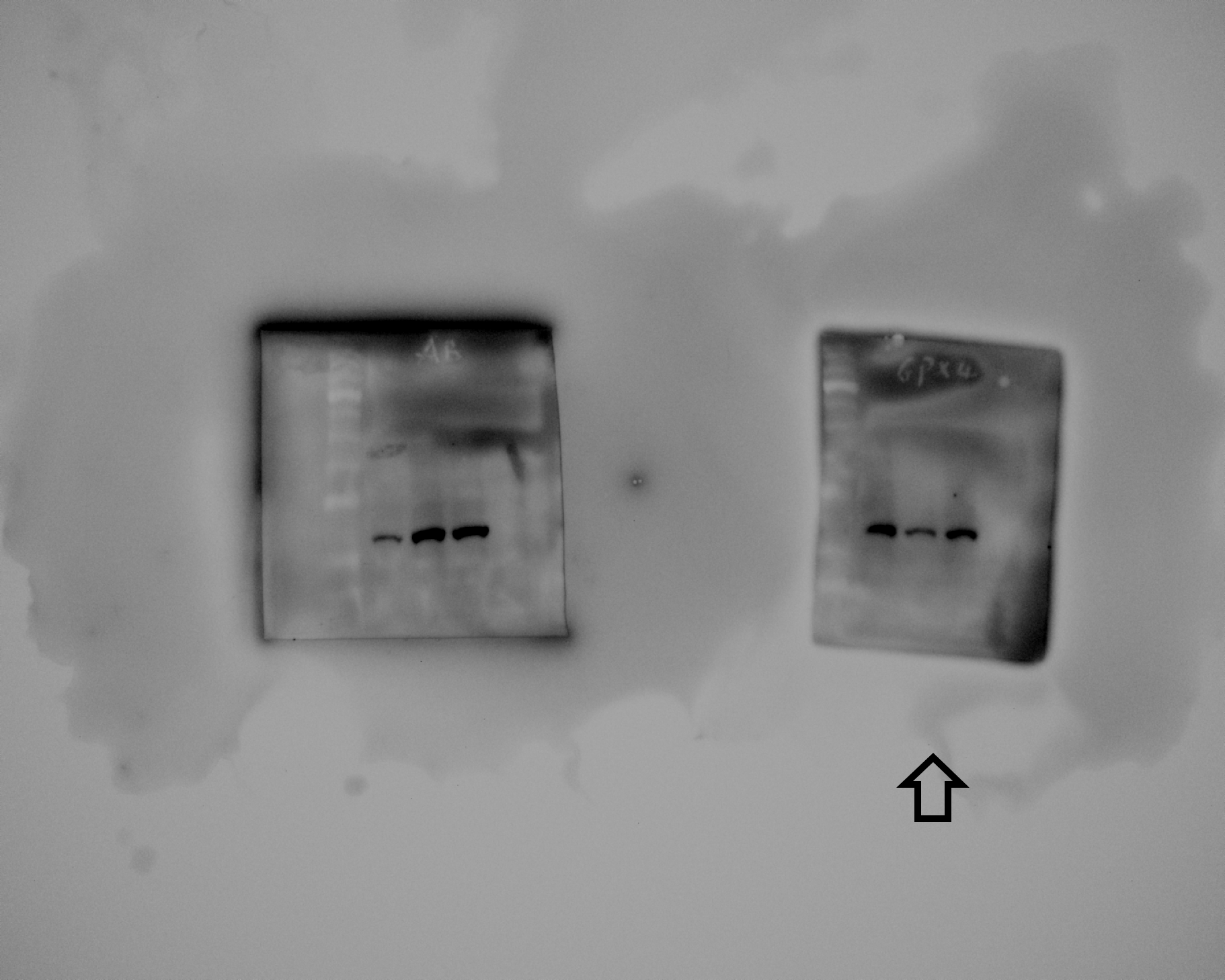

Supplement: Supplementary file 1 [file DataSheet1.ZIP › Original data/Figure 4 (D) GPX4/GPX4.tif]

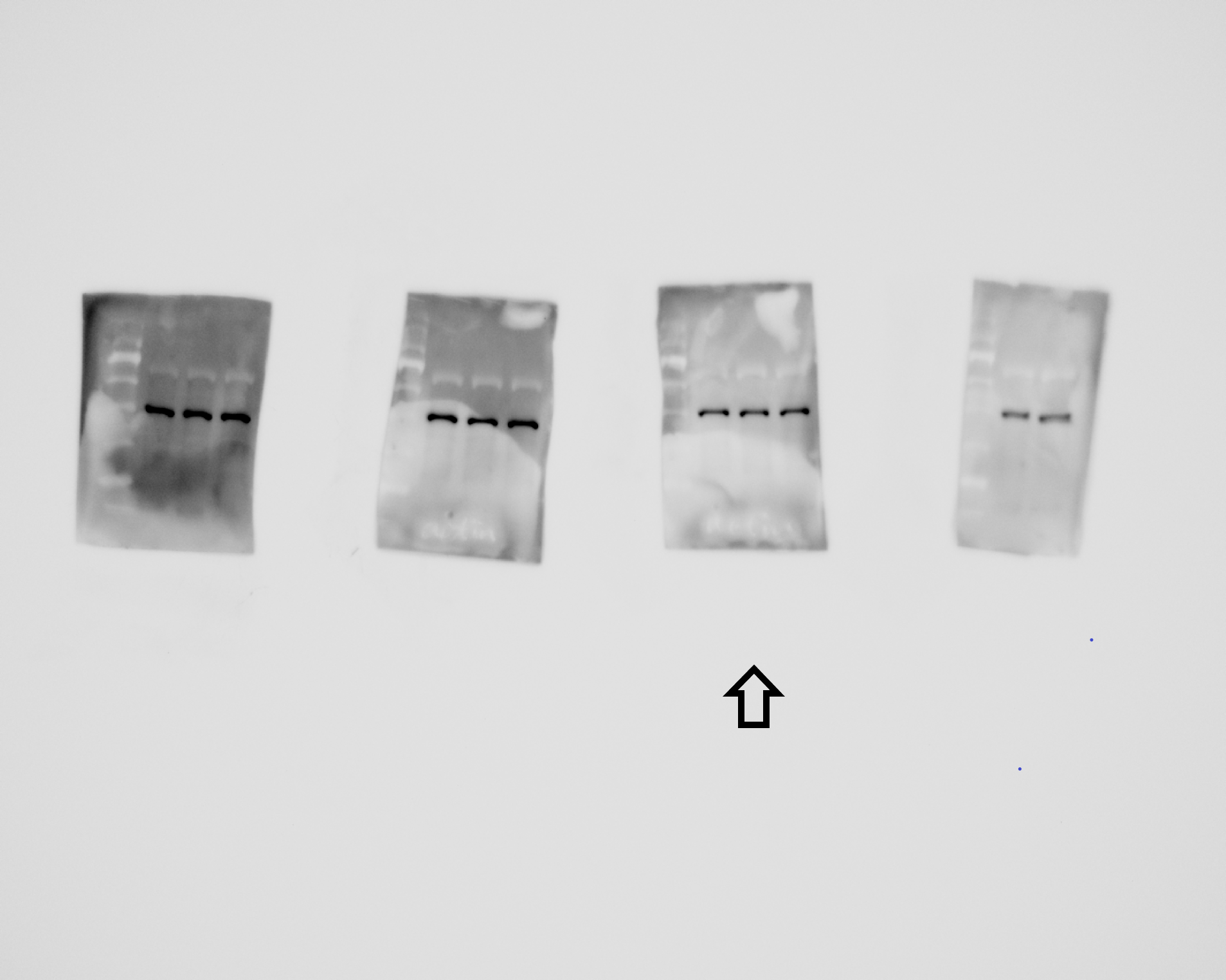

Supplement: Supplementary file 1 [file DataSheet1.ZIP › Original data/Figure 4 (D) GPX4/actin.tif]

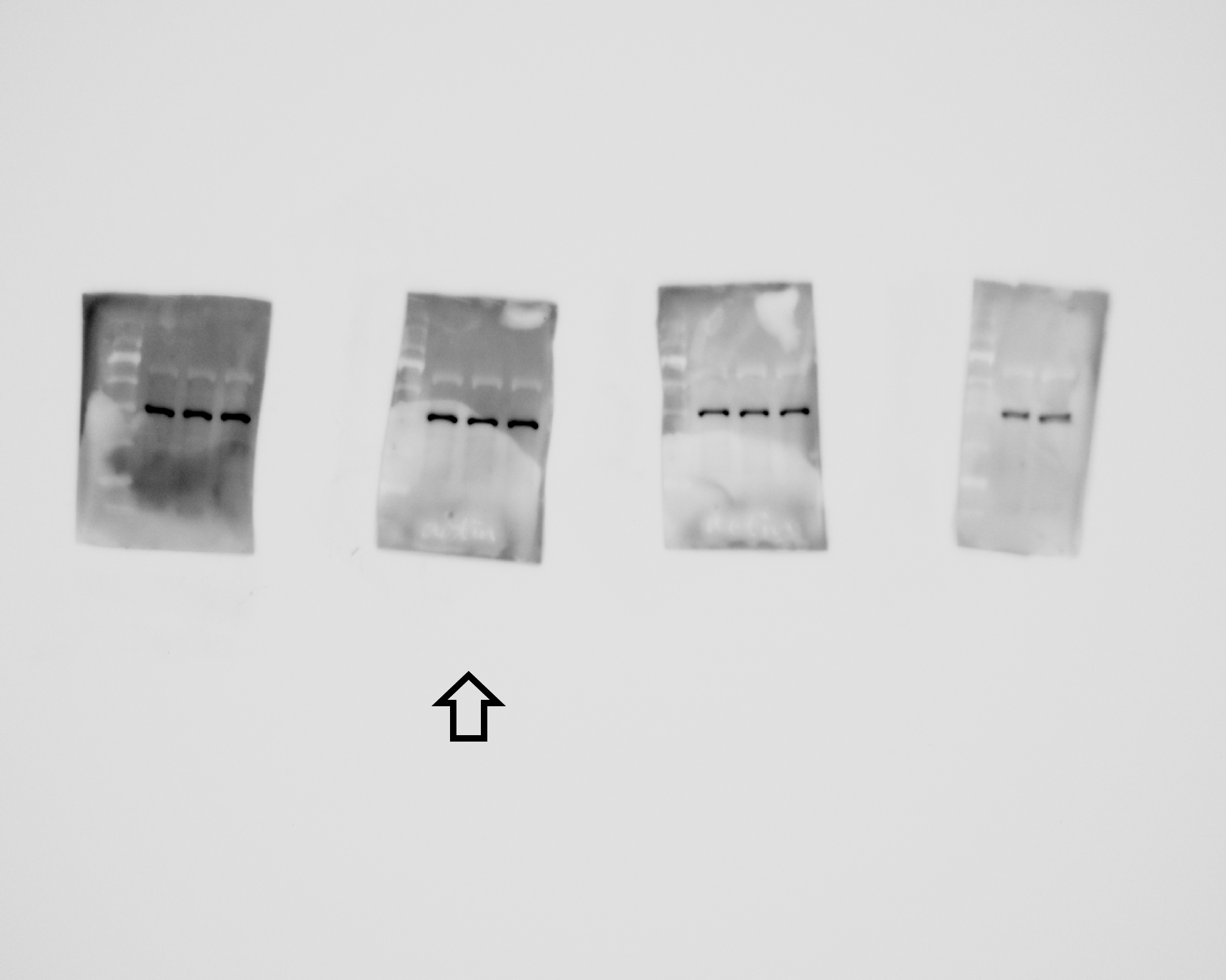

Supplement: Supplementary file 1 [file DataSheet1.ZIP › Original data/Figure 5 (A) tau, p-tau/actin.tif]

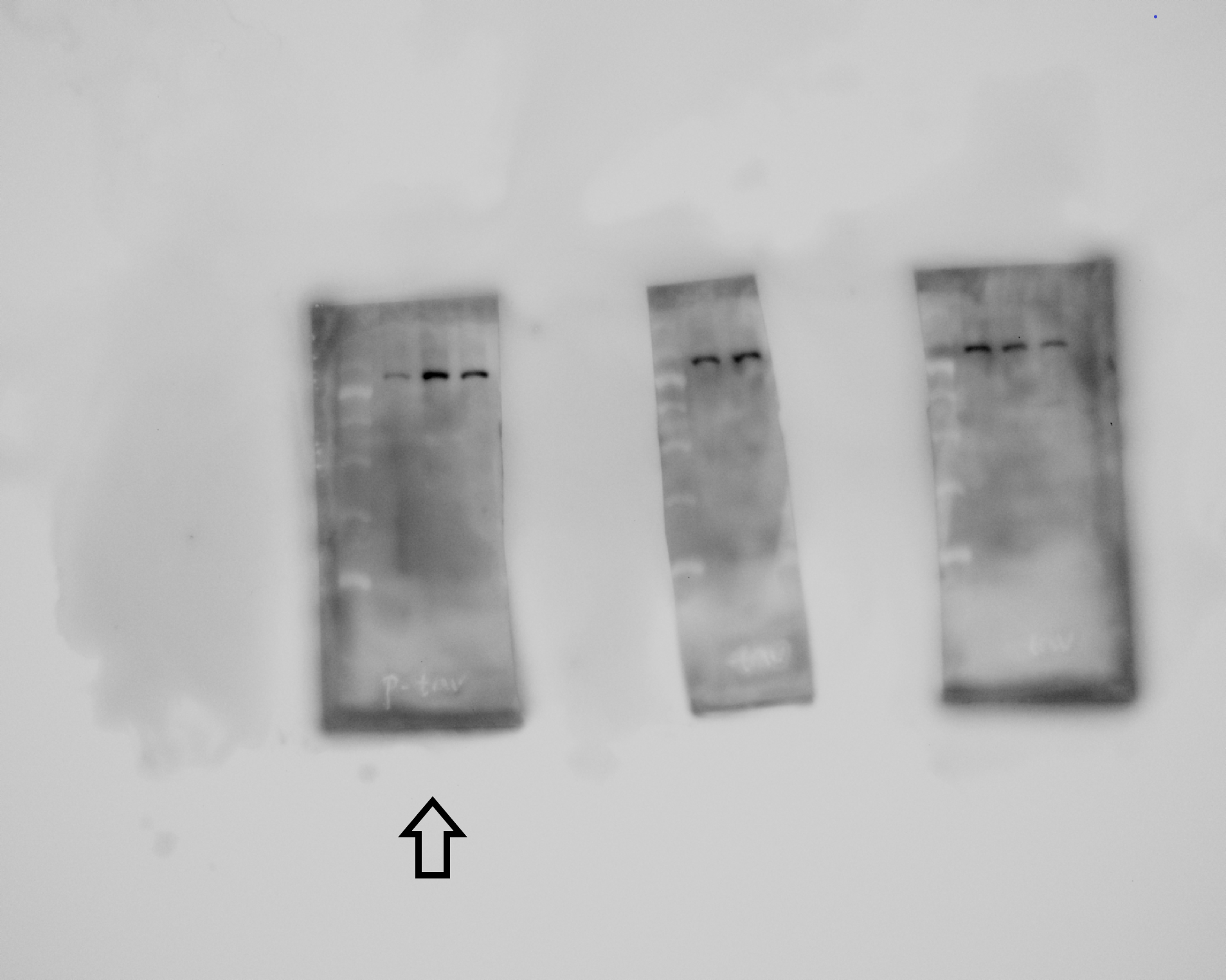

Supplement: Supplementary file 1 [file DataSheet1.ZIP › Original data/Figure 5 (A) tau, p-tau/p-tau.tif]

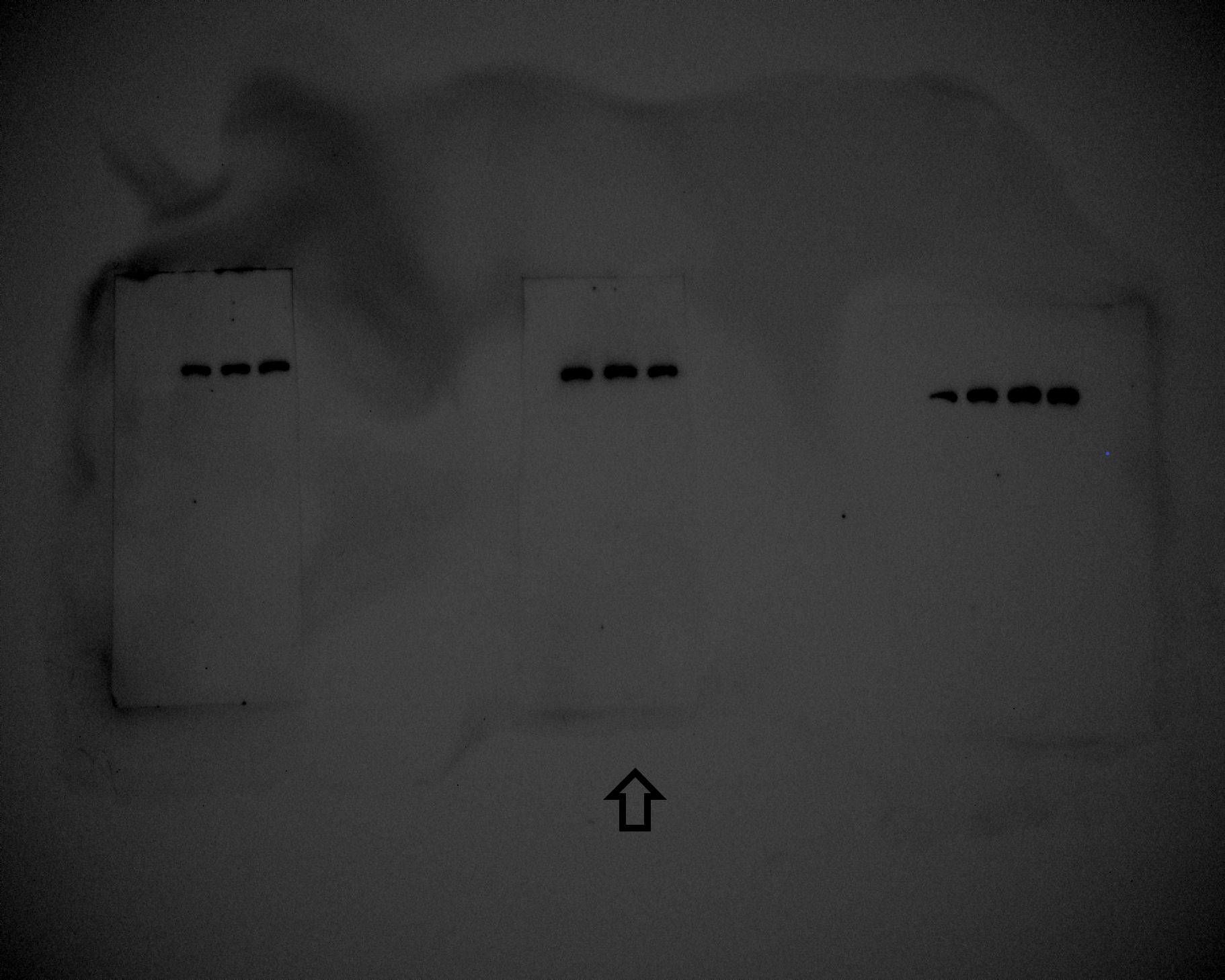

Supplement: Supplementary file 1 [file DataSheet1.ZIP › Original data/Figure 5 (A) tau, p-tau/tau.tif]

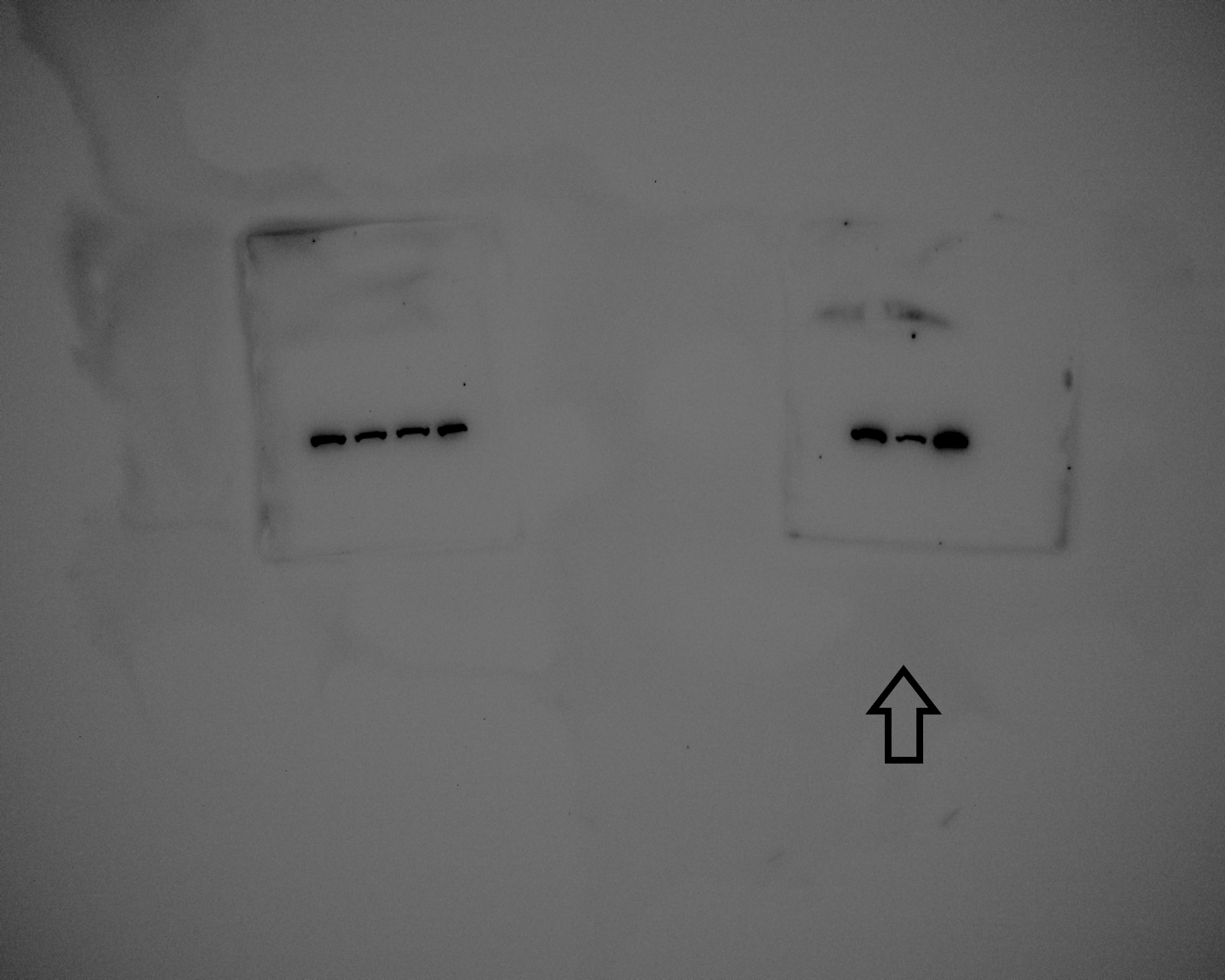

Supplement: Supplementary file 1 [file DataSheet1.ZIP › Original data/Figure 5 (C) iron, ROS, GPX4/GPX4.tif]

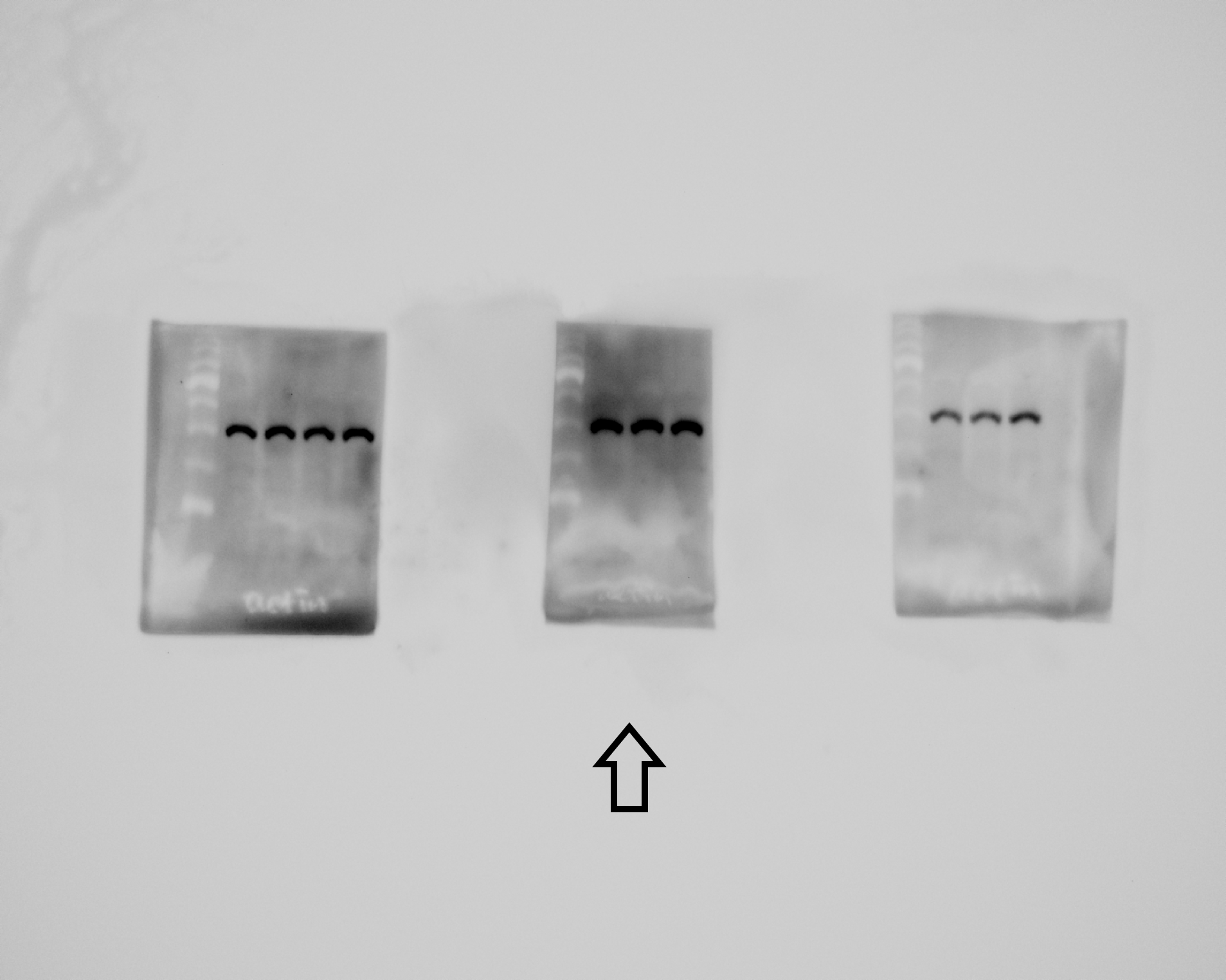

Supplement: Supplementary file 1 [file DataSheet1.ZIP › Original data/Figure 5 (C) iron, ROS, GPX4/actin.tif]
